# New Subscription Scheme Confirmed

**Published:** 1920-11-27

**Authors:** 


### THE COLLEGE OF NURSING (Limited by Guarantee).

### New Subscription Scheme Confirmed.

### (Verbatim Report.)

THE Second (Confirmatory) Extraordinary General Meeting was held at the Home of the Royal Society of Medicine, 1 Wimpole Street, London, on Saturday, November 20, 1920, at 3 o'clock, the Hon. Sir Arthur Stanley in the chair.

The CHAIRMAN: I ask the Secretary to read the notice convening the meeting.

The SECRETARY (Miss Rundle) read the notice.

The CHAIRMAN: I now ask the Secretary to read the minutes of the last meeting.

The SECRETARY read the minutes, which were approved and signed.

The CHAIRMAN: Ladies, this is the confirmatory meeting, according to the Acts. After you have had an extraordinary general meeting—of which you have just heard the minutes —you have to have a confirmatory meeting a fortnight afterwards. This is that confirmatory meeting. It is generally taken as practically a formal meeting, and I think, unless anybody has got any criticism to make, or an amendment to suggest, that will probably be the course you wish to adopt now. Therefore I formally call upon Miss Biggar to move the first resolution.

Miss BIGGAR: I have pleasure in proposing that the resolution, as read, be adopted.

Miss STEPHENSON: I have much pleasure in seconding it. Carried.

The CHAIRMAN: I call upon Miss Crawford to propose the second resolution.

Miss CRAWFORD: I have much pleasure in proposing the second resolution.

Miss BOWYER: I have much pleasure in seconding it. Carried.

The CHAIRMAN: That is the formal part of the business. The result of that now is, taken in conjunction with the resolution of the Council, which has the power of fixing the annual subscription, that new members from November 20 onwards will pay, in addition to their guinea, which is the entrance fee to the College, an annual subscription of five shillings. We have consulted those Associations which are most like this one to find out what they have found best in the way of collecting the subscriptions, and we find the best way is that new members joining will pay their five shillings subscription and their one guinea entrance fee when they join, and they will be called upon to pay the five shillings every year on the same day of the year as they paid their first subscription. That is to say, suppose a new member joins in December this year, she will be called upon to pay her guinea when she joins and the five shillings, and she will be called upon next year to pay her five shillings subscription, and so on each year. Reminders will be sent out a month before the subscription becomes due, so that none can possibly claim that they did not know they ought to pay the five shillings. That is as regards new members. We can only insist on those new members who join after November 20 paying the annual subscription, but we have asked our existing nineteen thousand odd members to pay a voluntary contribution. There are two ways in which they can pay their voluntary contribution, one is by becoming life-members on payment of five pounds. Any existing members who pay five pounds can rest secure for the remainder of their lives, for they will never be called upon for anything else, because we cannot do it. If they pay five pounds now, that franks them for the rest of their lives, and they will have no annual subscription to pay. Then there are those of our members who may not wish to bind themselves to pay five shillings annually, but we hope that those members will give us what they can. There will be a certain number of our present members who will agree to pay five shillings annually as if they had become new members after November 20. We shall send out regular forms to be filled up. Those members who wish to become annual subscribers will be asked to pay their five shillings annually in the month of November. We mention that month because it will enable us to get in the bulk of our subscriptions conveniently before the end of the year—that is, before the Christmas bills come in. If I have not made it quite clear to all what the position is I shall be very grateful if you will ask questions; we want this to go out in such a way that nobody can possibly make any mistake about it. What Miss Rundle wants me to make clear is, that all the existing members of the College—that is, all who have joined by to-day, are already life-members in fact; they have paid their guinea, and we cannot force them to pay any more. If any of you like to become a sort of super-life-member— (laughter)—by paying another five pounds we will accept it very willingly, then you will feel you are paying your five shillings a year without any trouble to yourselves. The rota will be distributed quarterly to all members, as at present, and that will be done free of charge. We feel it is absolutely essential that in a Society like this our members shall know, as far as we can possibly manage it, exactly what is going on. We shall, therefore, give it away free of payment. Our hope is that we shall be able, out of our advertisements, and so on, to make it a self-paying concern; it has not arrived at that happy position yet. You can help us to get advertisements by patronising the firms who advertise, and in that way you can do something towards the support of the College.

I now wish to propose a vote of thanks to the Council of the Royal Society of Medicine for lending us this Hall for the meeting on November 4 and to-day. I have very great pleasure in proposing that. We could not possibly have a better meeting-place than this Hall, and it is a great thing for us to know we are meeting with the approval and the generous support—for they lend us the Hall free—of the profession. I hope that in a very short time we shall have our place next door, which will belong to the College.

Miss TURNBULL: I have much pleasure in seconding it. Carried unanimously.

